# F82. COGNITIVE BIASES IN PATIENTS WITH SCHIZOPHRENIA AND HIGH SCHOOL STUDENTS: ASSOCIATION WITH PSYCHOPATHOLOGICAL SYMPTOMS

**DOI:** 10.1093/schbul/sby017.613

**Published:** 2018-04-01

**Authors:** Francesc Estrada, Maribel Ahuir, Josep Maria Crosas, Claudia Vilaplana, Alba González-Fernández, Meritxell Tost, Irina Olasz, Nora Mesa, Estefania Gago, Montserrat Pàmias, José Antonio Monreal, Diego Palao, Javier Labad

**Affiliations:** Parc Taulí Sabadell

## Abstract

**Background:**

Some cognitive biases, mainly the “jumping to conclusions” and attributional styles, play a key role in the formation and maintenance of delusions. Other thinking errors include dichotomous thinking, emotionally based reasoning, and catastrophising. The aim of our study was to assess the relationship between cognitive biases and psychopathological symptoms (positive, negative, depressive) in a clinical sample of patients with schizophrenia and a population sample of high school students.

**Methods:**

The clinical sample included 35 patients with schizophrenia (35.6 ± 10.8 years, 40% women) attending to the Department of Mental Health from Parc Taulí Hospital Universitari (Sabadell, Spain) and 45 high school students (16.6 ± 0.9 years) located in the same province.

Cognitive biases were assessed with the Cognitive Biases Questionnaire, that covers 5 types of biases (intentionalising [I]; catastrophizing [C]; dichotomous thinking [DT]; jumping to conclusions [JTC]; and emotional reasoning [ER]) and also gives a total score. Psychopathological symptoms in patients were assessed with the Positive and Negative Syndrome Scale (PANSS) and the Calgary Depression Scale for Schizophrenia (CDSS). Psychopathological symptoms in high school students were assessed with the Community Assessment of Psychic Experiences (CAPE).

Statistical analyses were performed with SPSS version 21.0. CBQ scores between groups were compared with Student’s T-test. The association between dimensions of the CBQ and scores of psychopathological scales was tested with Spearman’s correlations. Significance level was set at p<0.05 (two-tailed).

**Results:**

CBQ total scores did not differed between patients with schizophrenia (45.3 ± 8.2) and high school students (44.2 ± 6.7). No significant differences between groups were found in any of the five cognitive biases.

When exploring the relationship between cognitive biases and psychopathological symptoms in patients with schizophrenia, total CBQ scores were associated with CDSS scores (r= 0.65, p<0.001). In relation to particular cognitive biases, depressive symptoms were associated with all cognitive biases (I: r= 0.43, p= 0.017; C: r= 0.62, p<0.001; DT: r= 0.42, p= 0.020; JTC: r= 0.46, p= 0.012; ER: r= 0.57, p= 0.001), positive symptoms with ER (r= 0.43, p= 0.009) and general psychopathology symptoms of the PANSS with C (r= 0.34, p= 0.044), DT (r= 0.35, p= 0.041) and ER (r= 0.45, p= 0.007).

In high school students, CBQ total scores were associated with positive (r= 0.43, p= 0.003) and depressive (r= 0.35, p= 0.020) symptoms. In relation to particular cognitive biases, depressive symptoms were associated with DT (r= 0.47, p= 0.001) whereas positive symptoms were associated with DT (r= 0.31, p= 0.036) and ER (r= 0.30, p= 0.047).

**Discussion:**

Although we did not find significant differences in the presence of cognitive biases when comparing two different samples, similar associations were found when exploring the relationship between cognitive biases and psychopathology symptoms. Our results are in accordance previous studies reporting the role of some cognitive biases on the risk of developing psychotic symptoms. On the other hand, a clear association between cognitive biases was found for depressive symptoms in both patients with schizophrenia and high school students. Our study highlights the importance of identifying and treating cognitive biases with appropriate therapies (e.g. metacognitive training) for improving the outcome of psychoses in both patients and people at risk for developing a psychotic disorder in the future.

